# Precision genome engineering in rice using prime editing system

**DOI:** 10.1111/pbi.13395

**Published:** 2020-06-17

**Authors:** Kai Hua, Yuwei Jiang, Xiaoping Tao, Jian‐Kang Zhu

**Affiliations:** ^1^ Shanghai Center for Plant Stress Biology CAS Center of Excellence in Molecular Plant Sciences Chinese Academy of Sciences Shanghai China; ^2^ University of Chinese Academy of Sciences Beijing China; ^3^ Department of Horticulture and Landscape Architecture Purdue University West Lafayette IN USA

**Keywords:** prime editing, prime editor, Cas9, reverse transcriptase, rice

Dear editor,

Making precise changes in the genomes of organisms is challenging for most genome editing tools. Recently, a search‐and‐replace method, also known as prime editing, was developed that can introduce user‐defined sequence into a target site without requiring double‐stranded breaks (DSBs) or repair templates (Anzalone *et al.*, 2019). The prime editor contains a Moloney murine leukaemia virus reverse transcriptase (M‐MLV RT) fused to the C terminus of SpCas9 (H840A) nickase (Anzalone *et al.*, 2019). This fusion protein is guided by a prime editing guide RNA (pegRNA) to the target site. In addition to specifying the target site, the pegRNA contains a primer binding site (PBS) which is complementary to the PAM‐containing strand and template sequence for reverse transcription (i.e. RT sequence). The genetic information to be introduced into the target site is encoded in the RT sequence. The prime editors can introduce all 12 base‐to‐base conversions, precise small indels and their combinations. Therefore, they hold great promise for gene therapy as well as for precision breeding of crops. Here, we report the application of prime editors for precise genome engineering in rice plants.

We synthesized an engineered M‐MLV reverse transcriptase (D200N/L603W/T306K/ W313F/T330P) (Anzalone *et al.*, 2019) and used it to construct the prime editor Sp‐PE2 and Sp‐PE3 for expression in rice (Figure 1a). Compared to Sp‐PE2, Sp‐PE3 can express an additional nick sgRNA (Figure 1a). To test whether the prime editors are functional in plant cells, we used a transgenic reporter to monitor their activity in rice calli. We constructed an expression cassette containing an inactive EGFP sequence driven by the CaMV 35S promoter (Figure 1b) and inserted it into Sp‐PE2 and Sp‐PE3. Both Y67 and G68, two essential chromophore residues in EGFP, were changed into stop codons (Figure 1b). Only two precise base conversions (T‐G and G‐C) can restore a wild‐type EGFP sequence, whereas indels or other forms of base conversions cannot. We designed a pegRNA with 13 nt PBS and 13 nt RT sequence targeting the inactive site in EGFP (Figure 1c) and loaded it into Sp‐PE2 and Sp‐PE3. The Sp‐PE3 contains an additional nick sgRNA that targets a site 47 nt away from the pegRNA‐induced nick. The T‐DNA vectors were introduced into rice calli through *Agrobacterium*‐mediated transformation. After integration into the rice genome, the expressed prime editors would edit the inactive EGFP sequence under the guidance of pegRNA.

**Figure 1:**
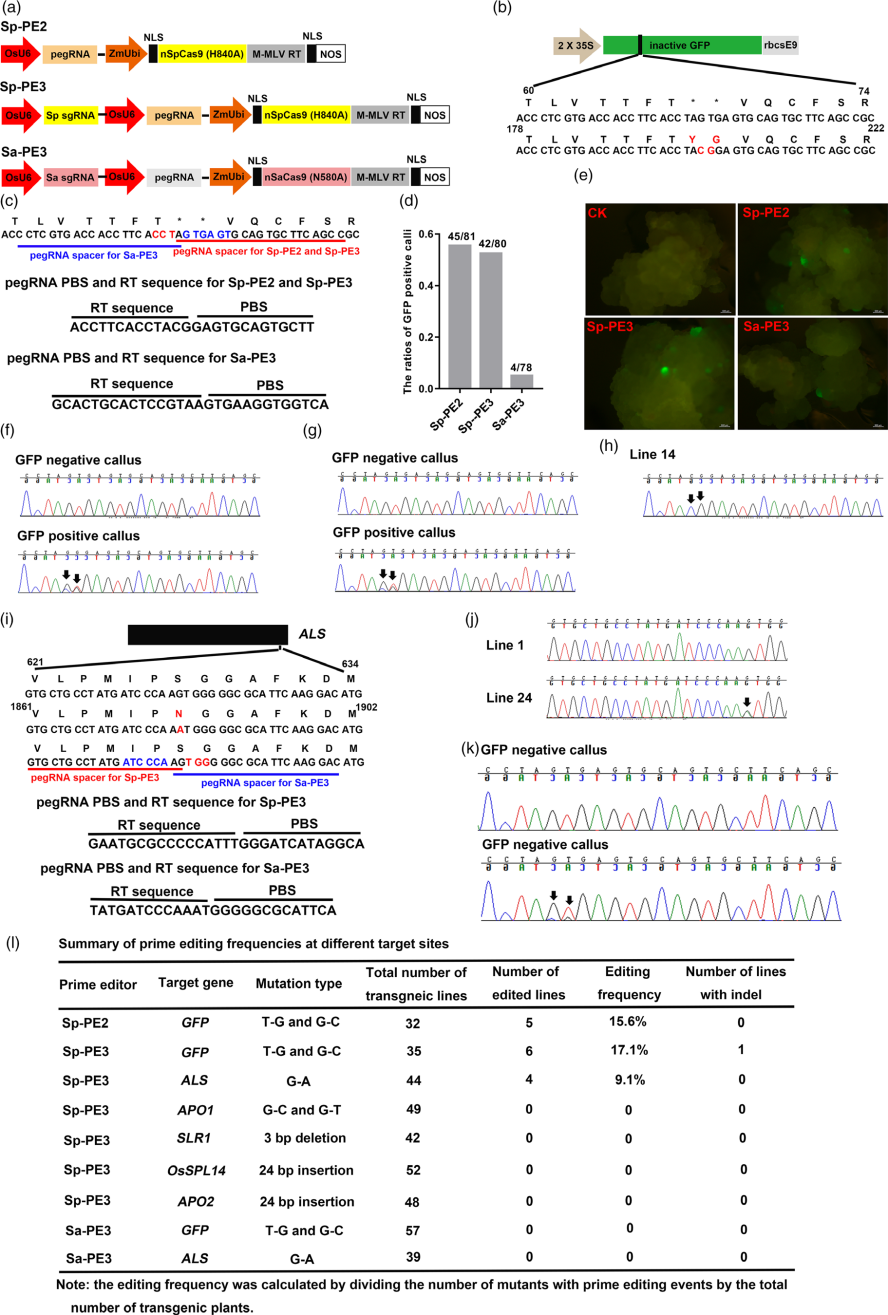
Developing a prime editing system for precision genome engineering in rice. (a) Schematic representation of different prime editors used in this study. (b) Diagram of the inactive GFP reporter. (c) The pegRNAs targeting the GFP inactive site designed for the different prime editors. (d) The ratios of GFP positive calli generated by different prime editors. (e) Detecting the GFP signal in transgenic rice calli under the fluorescence microscopy. In CK, the rice callus was not transformed by prime editors. Scale bars, 500 μm. (f) Sequence chromatograms of rice calli with or without GFP signals at GFP inactive site edited by Sp‐PE2. (g) Sequence chromatograms of rice calli with or without GFP signals at GFP inactive site edited by Sp‐PE3. (h) Sequence chromatogram of Line 14 at the GFP inactive site. This line was edited by Sp‐PE2. (i) The pegRNAs targeting ALS S627 site designed for prime editors Sp‐PE3 and Sa‐PE3. (j) Sequence chromatograms of Line 1 and Line 24 at ALS S627 site edited by Sp‐PE3. Line 1 was used as a control. (k) Sequence chromatograms of rice calli with or without GFP signals at GFP inactive site edited by Sa‐PE3. (l) Statistics of prime editing efficiencies at different target sites. PAM sequences recognized by SpCas9 or SaCas9 were marked by red or blue letters (c, i). Arrows point to the positions with an edited base (f, g, h, j, k).

After two weeks of selection, we found that more than fifty per cent of rice calli transformed by Sp‐PE2 showed GFP signals (Figures 1d,e). Sp‐PE3, which introduced a nick on the opposite strand, did not show an increased ratio of GFP positive rice calli (Figures 1d,e). After one month of selection, we randomly selected three hygromycin‐resistant rice calli with or without the GFP signal for genotyping and found that rice calli with GFP signals indeed harboured precise base conversions in the target site (Figures 1f,g). In contrast, rice calli without GFP signals did not show any mutation in the target region (Figures 1f,g). We then regenerated all hygromycin‐resistant calli (with or without GFP signals) and obtained 32 and 35 transgenic lines for Sp‐PE2 and Sp‐PE3, respectively. After genotyping, we found that the editing frequencies were comparable between Sp‐PE2 and Sp‐PE3 (15.6% and 17.1%) (Figure 1l). One line edited by Sp‐PE2 contained only the restored EGFP sequence (Figure 1h), and one line edited by Sp‐PE3 had no mutation at the pegRNA target site but had indels at the nicking sgRNA target region (Figure 1l). The rest of the Sp‐PE2 and Sp‐PE3 edited lines contained both the original and restored EGFP sequence.

To test whether the prime editor Sp‐PE3 can edit rice endogenous genes, we first chose the acetolactate synthase (*ALS*) gene as a target. A pegRNA containing 13 nt PBS and 16 nt RT template and a nick sgRNA 84 nt downstream of the site of the pegRNA‐induced nick were designed for *ALS* to introduce an S627N mutation, which makes rice plants resistant to imidazolinone herbicides (Figure 1i). We found that 4 out of 44 (9.1%) transgenic lines had a desired G‐A base transition at the target site and no indels were detected in any of the lines (Figures 1j,l). Among the four edited lines, two lines were heterozygous and the other two were chimeric. We then designed a pegRNA to introduce a C42F mutation in ABERRANT PANICLE ORGANIZATION 1 (APO1). However, no mutation was found at this site (Figure 1l). These results indicate that Sp‐PE3 can generate precise base conversions in rice but the efficiency varies at different sites. The slightly higher editing efficiency at the transgenic reporter may be due to a higher copy number of the inactive reporter gene in the rice genome. We also tested the ability of Sp‐PE3 to introduce precise small deletions and insertions into the rice genome. However, we could not found any prime editing events or even indel mutations at the targeted sites (Figure 1l).

The SaCas9 is a multiple‐turnover enzyme that releases the cleaved DNA products faster than SpCas9 does (Yourik *et al.*, 2019). We hypothesized that replacing the SpCas9 (H840A) in the prime editor by SaCas9 (N580A) may increase the prime editing efficiency because it may facilitate the reverse transcriptase to carry out reverse transcription. Therefore, we constructed Sa‐PE3 and designed the pegRNA targeting the inactive site of the EGFP reporter to test whether the Sa‐PE3 is functional in rice (Figure 1c). We found that 4 out of 78 rice calli showed very week GFP signals after two weeks of selection (Figures 1d,e). Genotyping the GFP positive calli indicated that only a small fraction of cells have a functional GFP sequence (Figure 1k). Consequently, we did not find any editing event from the regenerated stable transgenic lines (Figure 1l). Next, we designed a pegRNA to introduce an S627N mutation in ALS (Figure 1i). However, no transgenic lines showed any mutation at the target site (Figure 1l). Taken together, these results suggest that Sa‐PE3 has a lower editing activity at the two tested sites compared with Sp‐PE3. As the sequence and structure of sgRNA scaffold for SpCas9 and SaCas9 are different, the pegRNA structure for SaCas9‐PE3 may require further optimization to improve the editing efficiency.

In summary, the prime editing tool could precisely edit the transgenic reporter and endogenous gene in rice. During the preparation and review of our manuscript, several other prime editing systems in rice and wheat were reported (Li *et al.*, 2020; Lin *et al.*, 2020; Tang *et al.*, 2020; Xu *et al.*, 2020a; Xu *et al.*, 2020b). Tang and colleagues presented data from rice protoplasts only and did not obtain any stable transgenic lines with editing events (Tang *et al.*, 2020), whereas 1‐3 rice endogenous genes were edited with relatively low efficiencies in transgenic plants in the other studies. It is possible that the editing efficiency of the prime editing system can be improved by using other reverse transcriptases (Stamos *et al.*, 2017) or modifying the transgene selection system (Li *et al.*, 2019) to make the prime editors powerful tools for precision molecular breeding of crops.

## Conflict of interest

The authors declare no conflict of interests.

## Author Contributions

K.H., W.J., X.T. performed the experiments and analysed the data. K.H. wrote the manuscript. J.‐K. Z. supervised the project and edited the manuscript.
